# Response to Letter to the Editor: “The effect of chiropractic care on infantile colic: results from a single-blind randomised controlled trial” and “Identifying potential treatment effect modifiers of the effectiveness of chiropractic care to infants with colic through prespecified secondary analyses of a randomised controlled trial”

**DOI:** 10.1186/s12998-021-00385-2

**Published:** 2021-08-04

**Authors:** Lise Hestbæk, Lise Vilstrup Holm, Dorte Ejg Jarbøl, Henrik Wulff Christensen, Jens Søndergaard

**Affiliations:** 1 The Chiropractic Knowledge Hub, University of Southern Denmark, Campusvej 55, DK-5230 Odense M, Denmark; 2 Department of Sports Science and Clinical Biomechanics, University of Southern Denmark, Campusvej 55, 5230 Odense C, Denmark; 3 Research Unit of General Practice, Department of Public Health, University of Southern Denmark, J.B. Winsløws vej 9A, DK-5000 Odense C, Denmark

Firstly, we would like to thank Drs. Keil and Fludder for their interest in our study.

In Denmark, many children consult a chiropractor within their first year of life (19 % in 2020 [1]), and a large proportion of those consult due to excessive crying. Therefore, our intention was to design a pragmatic study, thus the treating chiropractor was free to treat the infant as considered optimal, reflecting normal practice. The only limitation to the autonomy of the treating chiropractors was the number of visits; both groups had to visit the clinic four times over a period of two weeks to limit attention bias and to improve blinding [2].

Thus, BK and CF are correct in pointing out that the results are not based on a specific predefined treatment; rather our results reflect those that can be expected in Danish chiropractic practice broadly.

BK and CF states “*The control group received an intervention likely to provide proprioceptive afferentation ……”*. While we cannot rule out that any handling, touching, or caring for an infant can affect the wellbeing, we fail to see that undressing and dressing the infant has an influence above standard everyday care. Further, if we understand them correctly, BK and CF also object to the treatment not necessarily including a thrust intervention, as this apparently should improve outcome (“*Increased neurophysiological responses occur with sub-100ms thrust duration compared to the non-thrust intervention”*). We are not sure how this relates to the first statement.

BK and CF mention an area of interest that is not covered in the present publications: which joints/areas were treated. We agree that this is a very interesting secondary analysis, and we hope to publish that in a subsequent publication.

Finally, they point out that “*The conclusion applies only to chiropractors without recognized post-graduate paediatric training.*” That is both correct and intentional. All chiropractors in Denmark are licensed to treat infants based on their university-based education. Although many of these chiropractors have an interest in paediatrics and have attended several seminars etc., only few have formalized post-graduate training. Therefore, we believe the choice of chiropractors increases the relevance to current practice and thus disagree with the statement that the study *“has minimal relevance to current practice and training”*. The situation might be different in Australia, and if there are specialized chiropractors broadly available across the country, we would welcome an opportunity to replicate the study among those.

## Data Availability

Not applicable
